# Risk factors, hematological and biochemical profile associated with colic in Delman horses in Gresik, Indonesia

**DOI:** 10.12688/f1000research.55312.2

**Published:** 2022-01-10

**Authors:** Muhammad Thohawi Elziyad Purnama, Dodit Hendrawan, Arya Pradana Wicaksono, Faisal Fikri, Agus Purnomo, Shekhar Chhetri

**Affiliations:** 1 Division of Veterinary Anatomy, Department of Veterinary Science, Faculty of Veterinary Medicine, Universitas Airlangga, Surabaya, East Java, 60115, Indonesia; 2 Animal Health Division, Indonesian Horse Veterinarian Association, Surabaya, East Java, 60115, Indonesia; 3 Division of Veterinary Physiology, Department of Veterinary Science, Faculty of Veterinary Medicine, Universitas Airlangga, Surabaya, East Java, 60115, Indonesia; 4 Department of Veterinary Surgery and Radiology, Faculty of Veterinary Medicine, Universitas Gadjah Mada, Yogyakarta, Yogyakarta, 55281, Indonesia; 5 Department of Animal Science, College of Natural Resources, Royal University of Bhutan, Lobesa, Punakha, 13001, Bhutan

**Keywords:** colic, Delman horses, domesticated animals, Gresik, hematological profiles, risk factors

## Abstract

**Background:** Horses are herd animals that have been domesticated in the last century. In several countries, an overview of risk factors and clinical evaluation in horses with colic has not been well‐described. This study aimed to evaluate risk factors and hematological profiles in horses associated with colic in Gresik, East Java, Indonesia.

**Methods:** A cross-sectional study was performed during April - October 2019. A total of 115 horses were diagnosed based on physical examination, clinical symptoms, and rectal examination. A questionnaire was asked to the horse-owners to analyze the risk factors while the clinical examination was performed and blood samples were collected for pre-treatment and 14 days post-treatment. Hematological profile was evaluated from a whole blood sample. Serum cortisol, plasma epinephrine, and norepinephrine concentrations were also evaluated after separating the aliquots.

**Results:** Of the 115 horses, 96 were diagnosed with colic. The horses with colic showed a significant association between cases with gender (p<0.021), breed (p<0.000), wheat bran feeding (p<0.015), concentrate feeding (p<0.003), anthelmintics administration (p<0.000), gastrointestinal parasites (p<0.000), dental diseases (p<0.024), previous exposure to colic (p<0.000), body condition score (p<0.000), and access to water per day (p<0.000). Based on whole blood and serum evaluation, there were ameliorated significantly on the hematological profile (p<0.01), serum cortisol (p<0.05), and plasma epinephrine (p<0.01) at 14 days post-treatment.

**Conclusion:** This study has identified factors associated with colic in Delman horses. The study provides crucial information to investigate cases of colic and to contribute the development of healthcare strategies during treatment and clinical evaluation.

## Introduction

Colic is defined as abdominal pain which is the most common cause of death in horses. Colic is categorized into two types: true colic caused by disorders of the digestive tract, and pseudo-colic due to disorders of organs other than the digestive tract. ^1^ True colic can be constipation colic (colon impaction), spasmodic colic (catarrhal enteralgia), tympanic colic (flatulent colic), and gastric colic (gastric distension) which are acute and implied to decrease horse performance and change habits. ^2^ Pseudo-colic can be caused by the presence of urolithiasis, uterine torsion, hepatitis, nephritis, myositis or tying up disease. ^3^ The severity of colic appears to vary from mild to severe based on its cause and treatment. The most common symptoms of colic are anorexia, sweating, restlessness, looking at the belly, kicking or biting the belly, spinning in the stable, scratching the legs, and rolling over. The appearance of common clinical symptoms usually cannot be distinguished between pseudo-colic and true colic. ^4^

There is a limited overview of the prevalence and cause of horses associated with colic in respective countries, particularly in areas that use the horse for transportation. Colic in horses was reported as a welfare issue and a crucial concern among horse-owners. ^5^ In previous studies, the incidence of colic in the working horse population was reported to be 30.4% in Ireland, ^6^ 36.8% in England, ^7^ 83% in Egypt, ^8^ 54% in Albania, ^9^ and 20% in Sweden. ^10^ Low quality hay feed, poor hygiene are increased risk factors for colic. The study found that 46.15% of hay samples contaminated with yeast and 30.76% contaminated with bacteria. ^11^ In addition, gastrointestinal parasites, high activity of horses and low access to water are the highest risk causes of colic. ^15^ Many horses with colic are associated with hemostatic disorders, both as a primary effect and as a complication of cardiovascular disorders. ^12^ Diagnostic procedures and pathophysiological mechanisms were studied in-depth to investigate coagulation disorders in horses. ^13^

In addition to identifying the history of the disease and performing a physical examination, evaluation of the hematological profile and blood chemistry of a horse can be done to trace the cause of the disease. The results of the examination can localize the cause of the disease based on the liver and spleen function. This method can evaluate hydration status, disease severity, inflammation, specific organ damage, endotoxemia probability, and determine disease prognosis. ^14^ This study was expected to provide an overview of colic risk factors and hematological profiles in horses with colic to monitor the progress of therapy during colic episodes and the possibility of recurrence.

## Methods

### Animals and ethical approval

Physical examination was performed according to standard operating procedures issued by the Indonesian Horse Veterinarian Association. This study was reported according to the Animal Research: Reporting of in vivo Experiments (ARRIVE) guidelines 2.0: author checklist. ^51^ All efforts were made to ameliorate any suffering of animals. The owner handles the horse by providing a sense of comfort to prevent stress, while the blood was collected according to standard examinations. Thus, a certificate of ethical clearance was not necessary for this study as the study did not affect normal animal behavior. Meanwhile, a permission letter (No.131/ADG/2019) was approved by Gresik Delman Association and informed consent was received by the horses' owner.

### Study period and locations

**Figure 1.**
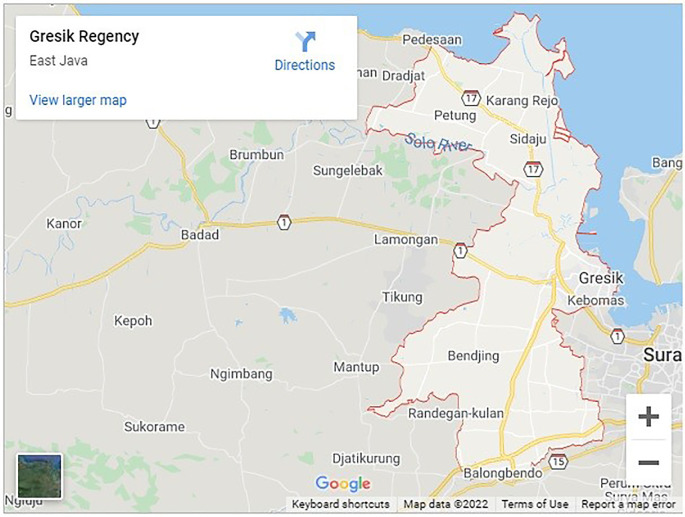
Map of Gresik Regency. [Powered by Google Map Source: https://goo.gl/maps/nZ5Aa1mhHqJkdGwe8].

This study was conducted for seven months (April - October 2019). The sample distribution was collected from Dukun (6°59′54.1′S 112°30′32.2′E) (n = 8), Panceng (6°55′42.0′S 112°27′58.0′E) (n = 21), Ujung Pangkah (6°55′10.2′S 112°32′46.7′E) (n = 19), Sidayu (6°59′33.1′S 112°33′44.6′E) (n = 15), Manyar (7°07′21.2′S 112°36′14.5′E) (n = 8), Kebomas (7°09′59.1′S 112°38′17.5′E) (n = 15), Menganti (7°17′34.9′S 112°35′07.9′E) (n = 8), Kedamean (7°19′20.5′S 112°33′57.6′E) (n = 10), and Driyorejo (7°21′11.1′S 112°37′43.9′E) (n = 11) ( Figure 1). The questionnaire was collected based on the owners' reporting. Serum and whole blood were evaluated at Gamma Scientific Biolab, Malang, East Java.

### Data collection

A cross-sectional study was designed to enroll Delman horses in Gresik, East Java, Indonesia. A total of 115 Delman horses at 3-11 years old and 300-400 kg weight were studied. The Delman horse as a riding animal was found in the owners' farm. The diagnosis was performed based on animal history, physical examination, clinical symptoms, and rectal examination. A questionnaire was asked to the owner to analyze the risk factors for colic horses. Questions were grouped into the following sections: age, season influence, gender, breed, wheat bran feeding, feeding on green fodder, concentrate feeding, anthelmintics, gastrointestinal parasites, dental disease, previous exposure to colic, body condition score, water source, access to water per day, musculoskeletal disease and bad vices. ^15^ Horse-owners completed the inquiries with one of the study team while the clinical examination was performed and blood samples were collected. In an attempt to validate owner-reported recurrent colic episodes, owners were asked to describe behavioral alterations and clinical signs that horses demonstrated during the colic episodes. ^16^

### Horse treatment

Colic therapy was performed according to current standard methods in the Indonesian Horse Veterinary Association. Lactated Ringers’ solution (Ringer Lactate, Widatra, Indonesia) was administered as initial treatment via jugular vein in all cases. Concurrently, Flunixin Meglumine (Flumine, Jaapharm, Mano, Singapore) was administered routinely in a dosage of 0.5 mg/kg, q 12 h, for three days. ^17^ The time before therapy (admission) was considered as pre-treatment. The next observation period was performed at 14 days as a post-treatment evaluation.

### Blood evaluation

For pre-treatment and 14 days post-treatment evaluation, blood from the jugular vein (10 ml) was collected into Vacutainer (BD ^®^, USA) with EDTA for measurement of hematological profile and plain tubes for serum isolation, respectively. All plain tubes allowed to clot for 10-20 minutes then centrifuged in a centrifuge machine (Hettich EBA 200 ^®^, GER), at a speed of 4000 rpm for 15 minutes. ^18^ The obtained serum was aspirated using a Pasteur pipette into a microtube and stored at −4°C. Serum cortisol was determined using ELISA method (My-Bio-Source ^®^, San Diego, CA, USA). ^19^ Meanwhile, for total protein, albumin, globulin and calcium were determined spectrophotometrically using Biuret method (Biolabo ^®^, France). ^20^

For hematological profile, whole blood samples were determined using the clinical chemistry analyzer (Hitachi 902 ^®^, Roche Diagnostics, USA). Each blood sample for evaluation of plasma epinephrine and norepinephrine concentrations was centrifuged immediately at 2500 rpm for 10 minutes, and plasma aliquots were analyzed using high performance liquid chromatography. ^21^

### Statistical analysis

All variables in the questionnaire were transformed into nominal criteria. A Chi-square test was performed to analyze independent data for each criterion associated with colic. The final analysis was done using logistic regression analysis with selected variables depending on p-values. Associations derived from conditional logistic regression were marked by odds ratio (OR) and relative risk (RR). A RR >1 reveals increased risk and a RR <1 reveals decreased risk.

Data of hematological and hormone evaluation were expressed as mean ± standard deviation (SD) then analyzed using one-way analysis of variance (ANOVA) followed by post hoc Tukey multiple comparisons test. Values were considered significantly different at p < 0.05. Statistical analysis was performed using SPSS v.25 software (IBM, Armonk, NY, USA).

## Results

### Clinical findings and risk factors

During the study period, 115 horses were examined and of these, 96 (83.48%) were diagnosed with colic. The colic diagnosis was based on the following typical clinical signs: abdominal pain, abdominal distention, dehydration, intestinal movement, intestinal sound, sense of appetite, heart rate per minute, profuse sweating, frequent urination, congested mucous membrane, and elevated body temperature ( Table 1).

**Table 1.** Distribution of colic clinical signs in Delman horses.

Variables	Colic ( *n* = 96)	%
Abdominal pain		
▪ Curling upper lips up	90	93.75
▪ Kicking at the belly	67	69.79
▪ Looking at the belly	81	84.38
▪ Paw at the ground	95	98.96
▪ Rolling	74	77.08
Abdominal distention		
▪ Absent	83	86.46
▪ Present	13	13.54
Dehydration		
▪ Mild	31	32.29
▪ Moderate	52	54.17
▪ Severe	13	13.54
Intestinal movement		
▪ Absent	19	19.79
▪ Constipation	56	58.33
▪ Diarrhea	21	21.88
Intestinal sound		
▪ Absent	54	56.25
▪ Present	42	43.75
Appetite		
▪ Off food	49	51.04
▪ Poor	34	35.42
▪ Good	13	13.54
Heart rate		
▪ <80 beat/min	41	42.71
▪ >80 beat/min	55	57.29
Profuse sweating	95	98.96
Frequent urination	82	85.42
Congested mucous membrane	89	92.71
Elevated body temperature	81	84.38

At the initial examination in the present study, all suspected horses were not classified based on the colic severity. Furthermore, according to the 96 questionnaires from colic horses and 19 questionnaires from normal horses, colic was associated with gender (p < 0.021; OR = 0.317), breed (p < 0.000; OR = n/a), wheat bran feeding (p < 0.015; OR = 0.180), concentrate feeding (p < 0.003; OR = n/a), anthelmintics administration (p < 0.000; OR = n/a), gastrointestinal parasites (p < 0.000; OR = n/a), dental diseases (p < 0.024; OR = n/a), previous exposure to colic (p < 0.000; OR = 32.250), body condition score (p < 0.000; OR = 0.022), and access to water per day (p < 0.000; OR = n/a) ( Table 2).

**Table 2.** Distribution of risk factors associated with colic in Delman horses.

Variables	Normal ( *n* = 19)	Colic ( *n* = 96)	SE	p-value	OR	RR
Age						
▪ <5 years	6	17	0.065	0.222	n/a	n/a
▪ 5-10 years	10	48				n/a
▪ >10 years	3	31				n/a
Season						
▪ Winter	9	53	0.093	0.531	0.730	1.175
▪ Summer	10	43				0.858
Gender						
▪ Male	9	71	0.043	0.021*	0.317	2.021
▪ Female	10	25				0.640
Breed						
▪ Sumba	9	89	0.041	0.000*	n/a	n/a
▪ Thoroughbred	9	5				n/a
▪ Mixed	1	2				n/a
Wheat bran feeding						
▪ No	2	38	0.045	0.015*	0.180	0.266
▪ Yes	17	58				1.481
Feeding on green fodders						
▪ No	16	89	0.026	0.230	0.419	2.165
▪ Yes	3	7				0.908
Concentrate feeding						
▪ <5 kg	0	32	0.042	0.003*	n/a	n/a
▪ >5 kg	19	64				1.500
Anthelmintics administration						
▪ No	0	75	0.045	0.000*	n/a	4.571
▪ Yes	19	21				n/a
Gastrointestinal parasites						
▪ No	19	22	0.045	0.000*	n/a	n/a
▪ Yes	0	74				4.364
Dental diseases						
▪ Absent	19	21	0.036	0.024*	n/a	n/a
▪ Present	0	75				1.280
Previous exposure to colic						
▪ Absent	15	10	0.039	0.000*	32.250	0.235
▪ Present	4	86				7.579
Body condition score						
▪ Poor	2	81	0.042	0.000*	0.022	5.726
▪ Good	17	15				0.125
Water source						
▪ Soft water	6	38	0.046	0.512	0.704	0.798
▪ Well	13	58				1.132
Access to water/day						
▪ Once	0	25	0.078	0.000*	n/a	n/a
▪ Twice	1	60				n/a
▪ Three times	12	8				n/a
▪ More than three times	6	3				n/a
Musculoskeletal diseases						
▪ Absent	19	88	0.024	0.192	n/a	1.091
▪ Present	0	8				n/a
Bad vices						
▪ Absent	15	85	0.032	0.257	0.485	1.837
▪ Present	4	11				0.892

Abbreviations: *n* = number of samples; SE = standard error; OR = odds ratio; RR = relative risk; n/a = not applicable.

In addition, based on RR score, an increased risk for colic was found for male horse (RR = 2.021), those administered wheat bran feeding (RR = 1.481), >5 kg of concentrate feeding (RR = 1.500), not performed anthelmintic administration (RR = 4.571), present of gastrointestinal parasites (RR = 4.364), present of dental diseases (RR = 1.280), colic recurrence (RR = 7.579), and poor body condition score (RR = 5.726) ( Table 2).

### Hematological profile, serum cortisol, and plasma catecholamine evaluation

Comparative results regarding the hematological profile of the normal and horse with colic during pre and 14 days post-treatment are presented in Table 3. In general, at 14 days post-treatment, the horses with colic improved significantly compared to pre-treatment according to blood parameters [TRBCs (p < 0.001), HB (p < 0.001), PCV (p < 0.001), MCV (p < 0.001), MCH (p < 0.001), MCHC (p < 0.01), neutrophil (p < 0.001), basophil (p < 0.001), monocyte (p < 0.01), and lymphocyte (p < 0.001)], clotting factors [Plt (p < 0.001), MPV (p < 0.001), PDW (p < 0.001), CT (p < 0.001), Prt (p < 0.001), Appt (p < 0.001), and fibrinogen (p < 0.001)], and other biochemical profiles [total protein (p < 0.001), albumin (p < 0.001), globulin (p < 0.001), and calcium (p < 0.001)]. The result was also revealed that all variables at 14 days post-treatment improved gradually similar to the normal group ( Table 3). The reference range ^22^ was also provided to add dynamic information on clinical evaluation during treatment of the colic episodes.

**Table 3.** Hematology profile and blood biochemistry at pre and 14 days post-treatment in Delman horses with colic.

Parameters	Ref ^22^	Normal ( *n* = 19)	Colic ( *n* = 96)	
			Pre-treatment	14 d post-treatment
TRBCs (× 10 ^6^)	6.5-12.5	10.7 ± 0.08 ^a,^***	9.6 ± 0.23 ^b^	10.7 ± 0.12 ^a,^***
HB (mg dL ^−1^)	11-19	13.4 ± 0.08 ^a,^***	14.4 ± 0.24 ^c^	13.6 ± 0.06 ^b,^***
PCV (%)	32.0-37.4	31.1 ± 0.59 ^b,^***	52.7 ± 0.58 ^c^	27.8 ± 0.45 ^a,^***
MCV (fl)	36-52	39.2 ± 0.08 ^a,^***	43.7 ± 0.59 ^b^	39.1 ± 0.13 ^a,^***
MCH (pg)	12.3-19.7	15.2 ± 0.14 ^a,^***	18.4 ± 0.09 ^c^	15.6 ± 0.19 ^b,^***
MCHC (g dL ^−1^)	34-39	335.5 ± 0.91 ^ab^	334.9 ± 0.63 ^b^	336.3 ± 0.12 ^a,^**
Eosinophil (× 10 ^3^ μL ^−1^)	0.0-0.9	0.8 ± 0.08 ^a,^*	0.9 ± 0.04 ^b^	0.8 ± 0.02 ^ab^
Neutrophil (× 10 ^3^ μL ^−1^)	2.7-6.7	6.4 ± 0.30 ^a,^***	8.9 ± 0.26 ^c^	7.1 ± 0.08 ^b,^***
Basophil (× 10 ^3^ μL ^−1^)	0.0-0.2	0.1 ± 0.01 ^a,^***	0.3 ± 0.02 ^b^	0.1 ± 0.01 ^a,^***
Monocyte (× 10 ^3^ μL ^−1^)	0.0-0.8	0.7 ± 0.06 ^ab^	0.7 ± 0.07 ^b^	0.6 ± 0.01 ^a,^**
Lymphocyte (× 10 ^3^ μL ^−1^)	1.5-5.5	5.3 ± 0.18 ^a,^***	3.4 ± 0.09 ^b^	5.2 ± 0.07 ^a,^***
Plt (×1)	100-600	568.4 ± 5.05 ^b,^***	256.9 ± 1.21 ^c^	590.6 ± 3.94 ^a,^***
MPV (fl)	9.0-10.2	8.9 ± 0.11 ^a,^***	13.3 ± 0.81 ^b^	8.4 ± 0.10 ^a,^***
PDW (%)	14.8-18.4	15.7 ± 0.34 ^b,^***	19.5 ± 1.19 ^c^	14.4 ± 0.12 ^a,^***
CT (min)	2.9-3.9	3.2 ± 0.05 ^a,^***	5.4 ± 0.08 ^b^	3.2 ± 0.04 ^a,^***
Prt (sec)	9.5-12.1	10.4 ± 0.29 ^a,^***	24.9 ± 0.42 ^c^	11.1 ± 0.46 ^b,^***
Appt (sec)	45.7-55.1	51.0 ± 0.59 ^b,^***	68.1 ± 0.73 ^c^	48.8 ± 0.34 ^a,^***
Fibrinogen (mg 100 mL ^−1^)	100-400	364.6 ± 5.31 ^b,^***	254.8 ± 10.43 ^c^	382.6 ± 0.87 ^a,^***
Total Protein (gm 100 mL ^−1^)	n/a	6.1 ± 0.04 ^a,^***	7.3 ± 0.17 ^b^	6.2 ± 0.05 ^a,^***
Albumin (gm 100 mL ^−1^)	n/a	2.9 ± 0.05 ^b,^***	3.8 ± 0.04 ^c^	2.7 ± 0.04 ^a,^***
Globulin (gm 100 mL ^−1^)	n/a	3.3 ± 0.05 ^b,^**	3.5 ± 0.05 ^c^	2.9 ± 0.09 ^a,^***
Calcium (gm 100 mL ^−1^)	n/a	11.4 ± 0.12 ^a,^***	7.8 ± 0.12 ^c^	8.7 ± 0.05 ^b,^***

Values are expressed in mean ± SD. One-way ANOVA was carried out followed by post hoc Tukey multiple comparisons test. Values are represented statistically when ^a,b,c,^ in comparison with normal group; *p < 0.05, **p < 0.01, ***p < 0.001, in comparison with pre-treatment group.

TRBCs = total erythrocyte count; HB = hemoglobin; PCV = packed cell volume; MCV = mean corpuscular volume; MCH = mean corpuscular haemoglobin; MCHC = mean cell hemoglobin concentration; Plt = platelets count; MPV = mean platelets volume; PDW = platelets distribution width; CT = clotting time; Prt = prothrombine time; Appt = activated partial thromboplastine time; n/a = not applicable (reference range not established).

**Figure 2.**
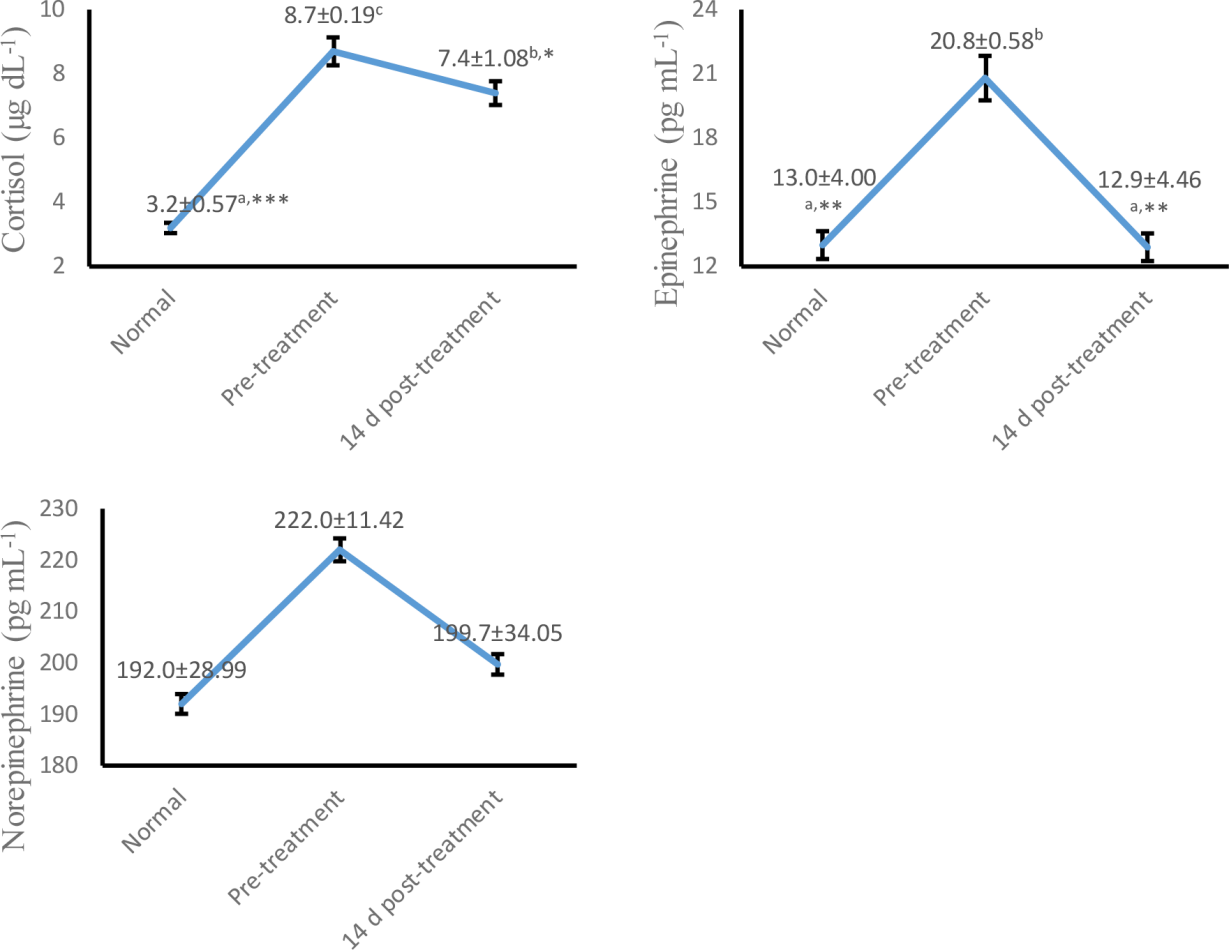
Trend concentrations of serum cortisol, plasma epinephrine and norepinephrine at pre and 14 d post-treatment in Delman horses with colic. Values are expressed in mean ± SD. One-way ANOVA was carried out followed by post hoc Tukey multiple comparisons test. Values are represented statistically when ^a,b,c,^ in comparison with normal group; * p < 0.05, ** p < 0.01, *** p < 0.001, in comparison with pre-treatment group.

As shown in Figure 2, serum cortisol (p < 0.05) and plasma epinephrine (p < 0.01) concentrations were decreased significantly at 14 days post-treatment compared to pre-treatment. However, plasma norepinephrine showed no significant difference (p > 0.05) at whole colic episodes. These findings indicate amelioration of the degree of sympathetic activation in horses associated with colic at 14 days post-treatment.

## Discussion

The current study presents the incidence and risk factors for the recurrence of colic in horses. Colic is still the main concern in horse management worldwide. In this study, the incidence of colic was higher in 71 male horses than 25 female horses ( Table 2). The incidence in Sumba horse was higher than other breeds, where 89 cases (92.7%) with colic were Sumba, five cases of Thoroughbred and two cases of mixed breeds ( Table 2). Male horses are widely used for traditional transportation. Meanwhile, female horses are only kept in the breeding area and are rarely used as working horses. ^23^ Sumba horses originated from the Sumbawa island and its known for having high movement and mobility. Sumba horses are commonly used as traditional transportation due to their ability to explore and survive in tropical environments. ^24^

The administration of wheat bran (58 cases) and >5 kg of concentrate feeding (64 cases) can increase the risk of colic especially if it does not appropriate the normal horse feed ratio ( Table 2). The procedure for feeding foals and five year old horses was equal. Horses in the growth period require quality raw materials for feeding, including protein with balanced amino acids for muscle development, contributing energy to the metabolic processes. Feed formula ratio consists of 60-70% concentrate. ^25^ Weaning horses are able to consume up to 3.5 kg of concentrate with 14-16% crude protein. The ratio of green fodder and concentrate is 30: 70 based on the dry matter. When a year old, horses need 13.5% crude protein with a green fodder ratio and a 40:60 concentrate of dry matter in the total ratio. Crude protein intake decreases to 11.5% at 18 months with a ratio of green fodder and 55:45 concentrate of dry matter ratio. Meanwhile, at the age of 24 months, crude protein needs reach up to 10% with a ratio of green fodder and 65:35 concentrate based on dry matter ratio. ^26^

In our study, 75 cases of colic horses without anthelmintic administration and 74 horses with administration were found to have gastrointestinal parasitic infections ( Table 2). Worm infections can occur through a single or mixed infection. Different types of infections occur in each animal due to differences in immunity to the worm infection. ^27^ Deworming leads a major role in controlling mixed infections. Horses infected with more than one type of worm may have a weak immune condition. Body endurance can be influenced by various factors such as nutrient intake, enclosure conditions, weather, anthelmintic administration, and the development factor of larval life in grasslands, i.e. climatic conditions, soil type, geographical location, and types of plants. High rainfall can also increase soil moisture. Humid conditions support infective larvae to survive. ^28^

There were 21 cases of colic horse with dental disease in our sample ( Table 2). Special treatment is needed due to the condition of the horses’ teeth, which are not suitable for chewing hard grass. Low lignin grass will be easily digested mechanically to prevent the risk of colic. ^29^ Lack of access to water per day can increase the risk of colic. In this study, horses received water access (per day) once in 25 cases, twice for 60 cases, three times for eight cases and more than three times for three cases ( Table 2). Horses must regularly get access to water to prevent colic at least eight liters every 6 hours. ^30^

In this study, 86 cases of horses had a history of colic, with 81 cases also having poor body scoring conditions ( Table 2). Poor body score condition and previous history of colic are correlated with recurrent colic cases. The aspects of the body score are influenced by daily nutrition, horse training, movement intensity, and anthelmintic administration. Horses who get regular dental and nail checks can improve animal behaviour. Horses with a history of colic should receive special treatment after feeding. Mild activity and exercise after feeding can reduce the risk of horse colic. ^31^

Cases of colic in horses can be associated as a cause of blood coagulation disorders. ^32^ It is triggered by an increase in blood concentration and a simultaneous decrease in coagulation factors. ^33^ Common symptoms of coagulopathy can be associated in colicky horses with gastrointestinal lesions, ischemia, inflammation, and peritonitis, which also depend on the severity of intravascular coagulation. ^34^ In the current study, the decrease in total platelet and fibrinogen at pre-treatment increased after 14 days post-treatment. In addition, there was an increase in coagulation time, activated partial thromboplastine time and prothrombine time at pre-treatment followed by a decrease after 14 days post-treatment. Thrombocytopenia, hypofibrinogenemia and decreased blood clotting time are reflected in the length of capillary refill time and petechial bleeding. Moreover, if the disorder is followed by inflammation as an activator of platelets which is released as an endogenous mediator. ^35^

Coagulation is disseminated in overlapping stages i.e. initiation, amplification, and propagation. ^36^ The initiation stage is characterized in cells that express tissue factor, which forms a complex with factor VIIa and activates factors IXa and Xa. Factor Xa binds to factor Va on the cell surface and produces a small amount of thrombin. Factor Xa is immediately inhibited so that it cannot move to other cells. ^37^ The amplification stage is characterized after exposure to colic, where platelets are released from the blood vessels, resulting in platelet attachment to the thrombin produced at the initiation stage. Thrombin activates platelets followed by surface changes and the release of partially active factor V. Thrombin also activates cofactors V and VIII, and activates factor XI to factor XIa. ^38^ The propagation step is performed on the surface of activated platelets. At this stage factor IXa binds to VIIIa, followed by an increase in the number of factor IXa as a result of platelet binding to factor XIa. The factor IXa/VIIIa complex activates factor Xa on platelets and immediately binds to factor Va thereby converting prothrombin to thrombin, followed by the thrombin complex converting fibrinogen to fibrin. ^39^ In another study, it has been reported that prolonged activated partial thromboplastine time and prothrombine time are the most frequently observed abnormalities in the coagulation profile of the colicky horse. ^40^

This study showed an increase in total protein, albumin, and globulin in the pre-treatment period. Several colicky horses with symptoms of dehydration, hemoconcentration, and acute loss of consciousness showed similar results with an increase in total protein. ^41^ In addition, decreased calcium levels are associated with tremor and muscle paralysis during episodes of colic. ^42^ Calcium also plays a role in the coagulation stage, in particular being positively correlated with fibrin activity. ^43^ The duration of the disease is related to the increase in plasma fibrinogen, serum albumin concentration and WBC count. Plasma fibrinogen concentrations and total WBC were significantly increased when evaluated as a single variable. Serum amyloid A (SAA) concentration showed a better improvisation as a marker for the duration of colic. This is due to the long interval between pre and post colic investigations and the support of early symptom information from horse owners. ^44^

Meanwhile, serum cortisol and plasma epinephrine levels were detected to be elevated in colic episodes in our study. This activity is predicted to be associated with an increase in plasma lactate concentration, blood pH, heart rate, and hemodynamic disorders. ^45^ The severity of septic shock following lactic acidosis has the potential to induce acute endotoxemia and deteriorating the condition of the horse during episodes of colic. High levels of plasma epinephrine can carry a risk of death. ^46^ In a previous study, horses did not survive if the plasma epinephrine concentration was <4 pg/mL at initial examination, whereas some colicky horses survived at a concentration of 222 pg/mL. ^47^ However, no definitive references have been reported for survivor horses in episodes of colic. The hemostatic response during tissue hypoxia and a decrease in mean arterial pressure are indicated to be the cause of the high plasma epinephrine concentration. ^48^ The increase in serum cortisol concentration during episodes of colic accumulates as a secondary result of increased secretion of the adrenal glands. ^49^ An indication of stress during colic episodes may be the common symptom of high serum cortisol concentrations, as shown in this study. In addition to our identified risk factors, the results of this study revealed improvements in hematological profiles, serum cortisol, and plasma epinephrine. We also emphasized information on the probability of coagulopathy and hemodynamic disorders during a colic episode.

## Conclusion

In conclusion, the main risk factors for colic in Delman horses in Gresik are gender, breed, wheat bran feeding, concentrate feeding, anthelmintics administration, gastrointestinal parasites, dental diseases, previous exposure to colic, body condition score, and access to water per day. Meanwhile, evidence of improved hematological profile, serum cortisol and plasma epinephrine were observed at 14 days post-treatment for colic. The results of this evaluation proved the improvement of the horses’ condition after therapy due to colic. This study may be used to inform future prospective studies investigating colic in working horse populations and to contribute effective preventative measures. In particular, it is necessary to evaluate the association of colic risk factors with working horses and gastric abnormalities.

## Data availability

### Underlying data

Figshare: Risk factor in horse with colic. https://doi.org/10.6084/m9.figshare.15148851.v1. ^50^

This project contains the following underlying data:

• Risk factor in horse with colic.xlsx
  • **Sheet 1.** Raw data for physical examination
  • **Sheet 2.** Raw data for risk factor questionnaires
  • **Sheet 3.** Raw data for hematological analysis

## Reporting guidelines

Figshare: ARRIVE checklist for ‘Risk factors and hematological profile associated with colic in Delman horses in Gresik, Indonesia’.

https://doi.org/10.6084/m9.figshare.16617511. ^51^

Data are available under the terms of the Creative Commons Attribution 4.0 International license (CC-BY 4.0).
