# Supplementary material for: Risk factors, hematological and biochemical profile associated with colic in Delman horses in Gresik, Indonesia
Source: F1000Res. 2022 Jan 10;10:950. Originally published 2021 Sep 21. [Version 2] doi: 10.12688/f1000research.55312.2 (PMC8787563; doi:10.12688/f1000research.55312.2)
Supplement: Peer review history

# Peer review: Reviewer response for version 2

**DOI:** 10.5256/f1000research.119273.r119244

**Published:** 2022-01-24

**Authors:** Muhammad Fiaz Qamar, Aziz ur Rehman

**Affiliations:** 1 Department of Pathobiology, "University of Veterinary and Animal Sciences sub Campus College of Veterinary and Animal Sciences", Jhang, Pakistan

The Research article has been updated and improved by the authors. All the comments have been addressed and answered. Now it is suitable for indexing.

Is the work clearly and accurately presented and does it cite the current literature?

Yes

If applicable, is the statistical analysis and its interpretation appropriate?

Yes

Are all the source data underlying the results available to ensure full reproducibility?

Partly

Is the study design appropriate and is the work technically sound?

Yes

Are the conclusions drawn adequately supported by the results?

Yes

Are sufficient details of methods and analysis provided to allow replication by others?

Partly

Reviewer Expertise:

Molecular and diagnostic parasitology

We confirm that we have read this submission and believe that we have an appropriate level of expertise to confirm that it is of an acceptable scientific standard.

# Peer review: Reviewer response for version 2

**DOI:** 10.5256/f1000research.119273.r119245

**Published:** 2022-01-18

**Authors:** Hamny Sofyan

**Affiliations:** 1 Laboratory of Anatomy, Faculty of Veterinary Medicine, Universitas Syiah Kuala, Banda Aceh, Indonesia

Generally, authors have been revised the manuscript according to reviewer’s suggestions. The discussion section has focused on the risk factors for the occurrence of colic in Delman horse. The relationship between the hematology profile and blood chemistry have been described in relation to the occurrence of colic. However, in some parts still need minor revision, although my decision is the manuscript was approved.

 There is still reference that is found in the results section, especially in the subsection, "Hematology Profile, Serum Cortisol, and Plasma Cathecolamin Evaluation". The sentence is, “ *the reference range* *^22^* *was also provided to add dynamic information on clinical evaluation during treatment of the colic episodes*”. Please move this sentence to the discussion section.

 Authors have been able to justify a non-stressful blood collection procedure in the revised manuscript. I think the statement “ *a certificate* *ethical clearance is not necessary etc*” should be removed.

Is the work clearly and accurately presented and does it cite the current literature?

Partly

If applicable, is the statistical analysis and its interpretation appropriate?

I cannot comment. A qualified statistician is required.

Are all the source data underlying the results available to ensure full reproducibility?

Yes

Is the study design appropriate and is the work technically sound?

Yes

Are the conclusions drawn adequately supported by the results?

Yes

Are sufficient details of methods and analysis provided to allow replication by others?

Yes

Reviewer Expertise:

Animal Anatomy and Physiology, immunohistochemistry

I confirm that I have read this submission and believe that I have an appropriate level of expertise to confirm that it is of an acceptable scientific standard.

# Peer review: Reviewer response for version 1

**DOI:** 10.5256/f1000research.58877.r94979

**Published:** 2021-12-03

**Authors:** Muhammad Fiaz Qamar, Aziz ur Rehman

**Affiliations:** 1 Department of Pathobiology, "University of Veterinary and Animal Sciences sub Campus College of Veterinary and Animal Sciences", Jhang, Pakistan

I have reviewed the manuscript and found minor gaps in the manuscript. The manuscript is well-written and clear to understand. A small number of typos and grammatical errors are present in the manuscript.

 Manuscript contains risk factors data associated with Colic in horses, some other risk factors can be added or addressed.

 The introduction section only describes the colic and its prevalence, the introduction should also contain the risk factors already reported and hematological alterations reported by researchers. Similarly, the prevalence parameter is missing in the methods section.

 The discussion section needs some improvements regarding objective and protein.

 The conclusion section can be improved by adding missing links for future studies.

Is the work clearly and accurately presented and does it cite the current literature?

Yes

If applicable, is the statistical analysis and its interpretation appropriate?

Yes

Are all the source data underlying the results available to ensure full reproducibility?

Partly

Is the study design appropriate and is the work technically sound?

Yes

Are the conclusions drawn adequately supported by the results?

Yes

Are sufficient details of methods and analysis provided to allow replication by others?

Partly

Reviewer Expertise:

Molecular and diagnostic parasitology

We confirm that we have read this submission and believe that we have an appropriate level of expertise to confirm that it is of an acceptable scientific standard, however we have significant reservations, as outlined above.

# Response

**Published:** 2021-12-23

**Authors:** Muhammad Thohawi Elziyad Purnama

**Affiliations:** Airlangga University, Indonesia

Dear Prof. Fiaz.

 I would like to thank you for your kind review of our manuscript.

 Regarding your judgment on our manuscript, we have added to the risk factor information gap in previous studies and the association with colic-relevant disease. We have added additional information on total protein as part of the discussion because it relates to the duration of the examination during colic. In the conclusion section, we have emphasized further research and the most important risk factors to be evaluated during the colic period.

 All the best wishes.

 Thank you.

# Peer review: Reviewer response for version 1

**DOI:** 10.5256/f1000research.58877.r94978

**Published:** 2021-10-14

**Authors:** Hamny Sofyan

**Affiliations:** 1 Laboratory of Anatomy, Faculty of Veterinary Medicine, Universitas Syiah Kuala, Banda Aceh, Indonesia

**General comments:**

 This research analyzed the risk factors for colic in Delman horses in Gresik, Indonesia and showed the success of the treatment based on hematological and biochemical profile and also catecholamine hormone levels. The result of this study can be used to decrease the incidence of colic in Delman horses in Indonesia.

**Specific comments:**

**Title:**

 Suggestion for title: Risk factors, hematological and biochemical profile associated with colic in Delman horses in Gresik, Indonesia

**Introduction**

 Author should pay more attention in this section. Author needs to highlight the researchable problem with colic in Delman horses, especially in Indonesia. Add the information about incidence rate of colic is in horses in Indonesia (if data is available).

**Methods**

 I think this study requires ethical clearance because there was a blood collection process and treatment was carried out in Delman horses with colic. Does your unit have ethics related to routine procedures performed on animals? If there is, I don't think ethical clearance is needed anymore.

**Results**

- I found discussion statement in the result section, please revised it.
- Suggestion: the title of Table 3: Hematology profile and blood biochemistry at pre and 14 days post-treatment in Delman horses with colic.

**Discussion**

- I think the discussion should be focus on the risk factors that significantly influence the occurrence of colic in Delman horses according to the results of this study and then compare with previous study.
- In this section, the relationship between hematology and chemical components of blood with the incidence of colic in Delman horse have not been clearly.
- Why are working horse not included in the variables of risk factors?

**Conclusion**

- “Meanwhile, evidence of improved hematological profile, serum cortisol and plasma epinephrine were observed at 14 days post-treatment for colic”. What is the meaning of this statement? Does this mean that the colic treatment has been suitable?
- Please, add the future improvements based on the research results in this section.

Is the work clearly and accurately presented and does it cite the current literature?

Partly

If applicable, is the statistical analysis and its interpretation appropriate?

I cannot comment. A qualified statistician is required.

Are all the source data underlying the results available to ensure full reproducibility?

Yes

Is the study design appropriate and is the work technically sound?

Yes

Are the conclusions drawn adequately supported by the results?

Yes

Are sufficient details of methods and analysis provided to allow replication by others?

Yes

Reviewer Expertise:

Animal Anatomy and Physiology, immunohistochemistry

I confirm that I have read this submission and believe that I have an appropriate level of expertise to confirm that it is of an acceptable scientific standard, however I have significant reservations, as outlined above.

# Response

**Published:** 2021-12-23

**Authors:** Muhammad Thohawi Elziyad Purnama

**Affiliations:** Airlangga University, Indonesia

Dear Dr. Hamny

 I would like to thank you for your kind review of our manuscript.

 We have improved the title and Table 3 heading according to your suggestion. Regarding the introduction section, we only found a colic report based on a repository and not yet published properly. 

 We have attached the ethical review along with the document (ARRIVE) guidelines 2.0: author checklist.

 We have also revised the results and discussion sections according to the findings of the investigated risk factors and compared them with previous study reports.

 As emphasized in the conclusion section, this study showed improvement in the condition of horses after treatment. Further studies need to examine the association of working horse risk with colic which is a limitation in this study.

 All the best wishes.

 Thank you.
